# An Exploratory Study of Over-the-Counter Medication Counseling Topics in Community Pharmacies and Alignment with Counseling Frameworks

**DOI:** 10.3390/pharmacy14010020

**Published:** 2026-02-02

**Authors:** Jason S. Chladek, Leena Jaiswal, Jamie A. Stone, Aaron M. Gilson, Taylor L. Watterson, Elin C. Lehnbom, Jukrin Moon, Emily L. Hoffins, Maria E. Berbakov, Michelle A. Chui

**Affiliations:** 1 Department of Pharmacy Administration and Public Health, St. John’s University College of Pharmacy and Health Sciences, Queens, NY 11439, USA; 2 Grossman School of Medicine, New York University, New York, NY 10016, USA; 3 Division of Social and Administrative Sciences, University of Wisconsin-Madison School of Pharmacy, Madison, WI 53705, USA; 4 Sonderegger Research Center for Improved Medication Outcomes, University of Wisconsin-Madison School of Pharmacy, Madison, WI 53705, USA; 5 Department of Pharmacy Systems, Outcomes, and Policy, University of Illinois Chicago College of Pharmacy, Chicago, IL 60612, USA; 6 Department of Pharmacy, Uppsala University, 752 37 Uppsala, Sweden; 7 Department of Industrial and Systems Engineering, University of Iowa, Iowa City, IA 52242, USA

**Keywords:** community pharmacy, over-the-counter, counseling, frameworks, medication safety

## Abstract

Community pharmacists can play an important role in patient safety by consulting patients on over-the-counter (OTC) medications. Several OTC counseling frameworks have been integrated into pharmacy education to guide pharmacists through these consultations, but limited work has been performed to examine how these frameworks are applied in real-world settings. The objective of this study was to identify the topics discussed during over-the-counter medication consultations and explore how they align with existing counseling frameworks. Participants were recruited from 10 community pharmacies. Participants were given hypothetical symptoms and asked to select OTCs for self-treatment. The selection process and potential interactions with pharmacy staff were recorded via Tobii Pro Glasses 2. Deductive and inductive content analysis of the recordings were used to compare participant–pharmacist consultations to existing OTC counseling frameworks. In total, 144 participants completed the study, with 32 (22%) having an OTC consultation with the pharmacist. Across all consultations, eight topic categories were identified. The consultations most frequently focused on discussions of product details and did not closely align with the OTC counseling frameworks. Future work should examine if and how this discordance contributes to OTC misuse among those interacting with pharmacists and potentially adapt or develop new frameworks to further support consultations and OTC safety.

## 1. Introduction

Community pharmacists play an important role in patient safety by ensuring that patients understand the medications they are using, including over-the-counter (OTC) medications, or drug products that patients can self-select and purchase without a prescription. As medication experts, community pharmacists can recommend OTC medications, as well as provide counseling on administration, dosing, and potential side effects [1]. To support this role, pharmacy education trains student pharmacists on how to engage in the Patient Care Process, a patient care framework developed by the Joint Commission of Pharmacy Practitioners (JCPPs). The goal of the Patient Care Process is to facilitate consistent delivery of patient care across the pharmacy profession. The “Collect” and “Assess” domains of this process assure that pharmacists collect and analyze necessary subjective and objective patient information relating to their medications to achieve optimal care [2].

In the United States and internationally, several OTC counseling framework mnemonics (e.g., QuEST, SCHOLAR-MAC, WWHAM, SIT DOWN SIR, and ENCORE) have also been integrated into pharmacy education to help pharmacists collect and assess relevant patient information (see Table 1 for framework details). These frameworks are intended to help future pharmacists triage patients and ensure that they are appropriately self-treating and correctly using OTCs. They also guide pharmacists in assessing self-care appropriateness, patient characteristics, symptom characteristics, and determining appropriate next steps [3,4].

These assessments can be especially important for vulnerable populations, such as older adults. Due to a combination of age-related physiological changes, multimorbidity, polypharmacy, cognitive decline, and general frailty, older adults represent a population particularly vulnerable to OTC-related adverse drug events (ADEs). Older adults are also among the highest consumers of OTC medications. Sleep aids and pain relivers, including aspirin, ibuprofen, acetaminophen, and diphenhydramine, as well as products containing these ingredients, are not only the most frequently used OTCs by older adults, but present the highest risk. These products have been frequently implicated in ADEs, resulting in emergency department visits [5,6,7,8,9]. In this context, applying structured OTC counseling frameworks may be especially valuable in helping older adults, as well as other vulnerable populations with similar risk factors, use OTC medications appropriately and safely.

Despite their widespread use in pharmacy education, limited work has been performed to examine how these frameworks are applied in real-world settings. Accordingly, the goal of this study was to identify the topics discussed during over-the-counter medication consultations in community pharmacies and explore how they align with existing counseling frameworks, with a particular focus on older adults and OTC products that are high-risk for this population. This knowledge can reveal how well these frameworks fit the existing work environment and whether additional measures are needed to optimize community pharmacist-provided OTC counseling, potentially reducing OTC misuse and related harm. Understanding this gap is a critical step to ensure that pharmacists are equipped to provide effective OTC counseling, which could directly impact patient safety and treatment outcomes.

## 2. Materials and Methods

### 2.1. Recruitment

Participants were recruited between June 2022 and October 2022 from 10 community pharmacies located within clinics of a large Midwestern health system in the United States. The community pharmacies were geographically dispersed to ensure engagement with diverse populations. Posters and fliers were available within the clinics to create awareness of the study. Patients who expressed interest approached researchers stationed outside the pharmacies and were recruited and screened for eligibility in real time. Eligible participants were ≥65 years old, would consider self-treating with an OTC, and passed a six-item cognitive impairment screener derived from the Mini-Mental State Examination (MMSE). The screener was administered in person by the trained research staff and assessed temporal orientation, attention, and three-item recall to identify potential cognitive impairment among participants [10]. The study was approved by the University’s Institutional Review Board.

### 2.2. Data Collection

After giving informed consent, participants were asked to select whether they had the most experience with self-treating cough/cold/allergy, pain, or sleep symptoms. Based on their selection, participants were then given a hypothetical symptom scenario. Participants were told to pretend that they had not taken any medications for the symptoms, and the symptoms were not bad enough to call a doctor.

Participants were fitted with Tobii Pro Glasses 2 and asked to go into the pharmacy to select one or more OTCs to treat the hypothetical symptoms. They were not given a time limit for this process and could interact with pharmacy staff if desired. To further help replicate a real-world experience, participants were given a study-designed (but inactive) credit card to “buy” their selected products. Although Tobii Pro Glasses 2 are capable of collecting eye-tracking data, they were solely used to audio- and video-record participants’ selection processes and any potential interactions with pharmacy staff [11]. Prior to data collection, pharmacy staff were instructed to interact with study participants as they typically would. The pharmacists were not explicitly told to consider OTC counseling frameworks during patient interactions.

Following this interaction, a 20 min phone interview was scheduled with the participant to collect demographic information. The interview guide was further detailed in a previous study [8]. After completion of this interview, participants were compensated for their time.

### 2.3. Data Analysis

Audio/video recordings were reviewed to identify consultations between participants and pharmacists during the OTC selections. Consultations could be initiated by either the patient or pharmacist but were only included in this analysis if the pharmacist assisted the patient in their decision-making and selection of an OTC product. Consultations were excluded if the pharmacist only greeted the participant or assisted them in checking out. Additionally, consultations were excluded if the pharmacist asked the participant if they had any questions, and the participant indicated they did not.

Recordings that met these criteria were reviewed by a primary coder (JSC) and a secondary coder (LJ) who performed deductive content analysis based on a priori topic categories derived from prominent OTC counseling frameworks (QuEST, SCHOLAR-MAC, WWHAM, SIT DOWN SIR, and ENCORE) [3,4]. These categories were chosen after identifying common themes across the frameworks. Table 2 shows how the a priori topic categories align with the frameworks. Table 3 shows example questions/statements that would be coded to each category. Counseling topics were included regardless of whether the patient or pharmacist mentioned it first. The coders independently performed inductive content analysis to identify new counseling topic categories outside of the a priori categories. All inductive findings, ambiguities, and questions were discussed during biweekly coder meetings. Frequencies of each counseling topic and specific product details discussed were calculated and presented below. Additionally, findings were mapped back to the original OTC counseling frameworks to determine which frameworks were most closely followed.

## 3. Results

In total, 144 participants completed the study, with 32 (22%) engaging in a consultation with a community pharmacist about their OTC selection. Overall, 13 (41%) participants selected the cough/cold/allergy symptom scenario, 17 (53%) selected the pain scenario, and 2 (6%) selected the sleep scenario. Of the 32 participants, 31 completed the follow-up phone interview. These 31 participants were an average of 72 years old. Overall, 29 (94%) participants identified as White, 2 (6%) as American Indian or Alaska Native, 1 (3%) as Black or African American, and 1 (3%) chose not to identify. No participants identified as Hispanic or Latino. A total of 16 (52%) participants identified as female, and 15 (48%) as male.

Overall, 24 (75%) of the consultations were initiated by the participants. Eight counseling topic categories were identified in participant–pharmacist interactions (see Table 4). In total, 28 participant–pharmacist consultations included discussions of “product details,” making it the most prevalent topic. Within these consultations, nine specific product details were identified, with frequencies shown in Table 5. Table 6 and Table 7 compare the identified topic categories by sex and selected hypothetical symptom scenario, respectively. Items in all tables are presented in the order of frequency, highest to lowest, to highlight the most commonly addressed topics.

The topic categories (and their frequencies) were mapped back to the five OTC counseling frameworks they were derived from. Assessment of self-care appropriateness, which is the first step in three of the five frameworks (QuEST, WWHAM, and ENCORE), was observed in only one consultation (3%). Symptom details and previous treatments are present in five and four (SCHOLAR-MAC, QuEST, WWHAM, and SIT DOWN SIR) of the OTC counseling frameworks, respectively. Yet, these topic categories only showed up in six (19%) pharmacist consultations each. Finally, discussions of product details and product recommendations were the most prevalent topic categories discussed, being identified in 28 (88%) and 19 (59%) of the consultations, respectively. However, “product details” only aligns with one OTC counseling framework (QuEST), and “product recommendation” aligns with two (QuEST, ENCORE). Notably, the two most prevalent topic categories both align with the QuEST framework. Only one topic category (discussion of cost–benefit) was identified via inductive analysis and was only noted in one consultation (3%).

## 4. Discussion

Only a minority of observed participants (22%) engaged in a consultation, highlighting that most OTC selections occurred without pharmacist involvement, let alone a consultation that comprehensively aligned with an existing framework. Study results showed that most consultations were initiated by participants. This makes sense, as community pharmacists typically do not have the capacity to approach every patient selecting an OTC. However, among both pharmacist- and participant-initiated consultations, there was a high level of discordance between the existing OTC counseling frameworks and the real-world interactions. Overall, the topics included in the consultations were highly limited and often did not align with the topic categories derived from the frameworks.

Among the five frameworks (QuEST, SCHOLAR-MAC, WWHAM, ENCORE, and SIT DOWN SIR), QuEST appeared to be the most closely aligned with what was observed in practice. This is largely because this framework emphasizes product recommendations and discussions of product details. However, the upstream components—such as assessing self-care appropriateness, symptom details, or previous treatments—were rarely addressed. This discordance occurred despite the fact that pharmacists often lack access to electronic health records and comprehensive patient information—a situation where adherence to these frameworks should be particularly helpful [12]. The results also suggest that pharmacists are taking a more responsive role when helping patients navigate OTCs and counseling on selections. In many cases, pharmacists would simply respond to the questions being asked, rather than proactively covering the topics included in the OTC counseling frameworks. Additionally, no major differences were noted between male and female participants or those selecting different symptom scenarios, though most participants selected the cough/cold/allergy and pain scenarios.

Several factors may help explain the observed patterns in the OTC consultations. First, community pharmacies are not only complex work system environments, but the role of community pharmacies has expanded to include increased responsibilities, such as vaccinations, medication therapy management, and health promotion and education programs [13,14]. Additionally, expanded scope of practice may lead to a more demanding workload [15]. In return, this may cause community pharmacists to deviate from existing guidelines or procedures, including those related to patient counseling [16]. Community pharmacy payment models may also pressure pharmacists to focus on responsibilities with greater financial incentives [17]. As a result, OTC counseling may be less of a priority.

A noteworthy point is that what is taught in pharmacy curriculum may not always directly translate to practice, creating a natural divide between the two. Additionally, this division may not always be clinically significant. For example, pharmacy students may be expected to closely follow these “high standard” OTC counseling frameworks during consultation simulations or formal assessments. However, pharmacists that use OTC counseling frameworks as guides and do not ask about every framework component due to external constraints may still effectively counsel patients on using OTCs. Furthermore, a more experienced pharmacist may appropriately triage patients based on patient cues, prior knowledge, or perceived risk, without the use of a guiding framework.

However, it is possible that discordance between OTC counseling frameworks and real-world consultations may translate to limited or insufficient counseling, which could be problematic. For example, the results of this study show that pharmacists did not consistently ask about symptom details, previous treatments, or current medications and health conditions. Instead, consultations largely focused on downstream discussions of product recommendations and product details. Overlooking these upstream assessments could potentially increase the risk of ADEs, especially when product recommendations are still being made. Notably, assessment of self-care appropriateness—the first step in several frameworks and a critical step to determine whether or not self-treatment is even appropriate—was rarely observed [3,4]. These points are especially important to consider given that the study focused on older adults, a population that not only frequently uses OTCs, but is exceptionally vulnerable to ADEs from the OTCs they use and higher-risk OTC products [8,9]. Even when product details were discussed with these individuals, the pharmacists often did not discuss side effects, interactions, or contraindications, potentially creating an even greater risk for ADEs. 

The goal of this study was to identify the topics discussed during over-the-counter medication consultations in community pharmacies and explore how they align with existing counseling frameworks. In addition to expanding future studies to include more pharmacies, patient populations beyond older adults, and additional symptoms, future investigation is needed to determine whether the discordance between real-world consultations and OTC counseling frameworks noted in this study is contributing to OTC misuse for those receiving consultations, as this study did not measure misuse. In other words, are structured, framework-based consultations necessary for preventing OTC-related problems and patient harm? If it is found that discordance between practice and frameworks significantly contributes to misuse or patient harm, additional work could be performed to better understand the factors leading to this discordance, including factors related to patient demographics or symptoms being treated, and how to best work around them.

Additionally, if discordance between real-world consultations and OTC counseling frameworks shows concern for OTC misuse and patient safety, potential work could also be performed to modify or develop new OTC counseling frameworks or tools for pharmacy education. Possible directions could include condensed lists that emphasize the most critical questions for ensuring safe OTC use. Frameworks or tools could also focus on enabling pharmacists to be proactive or leveraging patients as the drivers of the conversation by incorporating methods such as motivational interviewing [18]. Ultimately, adapted frameworks or new tools could help pharmacists ensure that patients are appropriately using OTC medications, allow for patient autonomy, and account for potential differences between pharmacy curriculum and real-world practice, including the demanding community pharmacy work environment.

### Limitations

There are limitations to this study that should be acknowledged. It was conducted within a single Midwestern health system and focused on a specific patient population. While both small and large pharmacies from urban and rural communities were included, the study was homogenous with respect to race and ethnicity. These factors may limit the generalizability to other geographic areas or populations. Participants addressed hypothetical symptoms from pre-selected options, rather than existing symptoms, possibly impacting participant–pharmacist interactions. The majority of participants selected the cough/cold/allergy and pain scenarios, limiting insights related to consultations for sleep products. Additionally, despite instructions to behave normally, pharmacists were not blinded and were aware they were interacting with study participants, which may have impacted their consultation process. Pharmacists were also aware of the participants’ general ages (≥65) and symptoms (based on the symptom scenarios), which could further influence interactions. In this study, it was unclear if pharmacists were previously familiar with the participants (health history, medication history, etc.), which could have affected their consultation process, and it was unknown which OTC counseling frameworks the pharmacists learned during training or had prior experience with. Pharmacists may have considered certain components of the frameworks internally, without verbally discussing them with the participants. Finally, we did not analyze differences in consultations based on pharmacist or pharmacy characteristics, nor did we explore the extent to which adherence (or the lack thereof) to OTC counseling frameworks influenced subsequent OTC use or misuse. As an exploratory study, these limitations highlight important next steps for future research, where these factors can be examined in greater depth among a more diverse sample.

## 5. Conclusions

By providing thorough patient counseling, community pharmacists are a promising resource for preventing OTC misuse and subsequent patient harm, especially for vulnerable patient populations such as older adults. However, this study showed that relatively few individuals engaged in OTC consultations with pharmacists, and when consultations did occur, the topics discussed did not comprehensively align with the existing frameworks designed to guide OTC counseling. Future work should examine if and how this discordance contributes to OTC misuse, including among those interacting with pharmacists, and potentially adapt or develop new frameworks to support consultations and OTC use.

## Figures and Tables

**Table 1:** OTC counseling frameworks [3,4].

Frameworks
**QuEST** Quickly and accurately access the patient Establish appropriateness for self-care Suggest appropriate self-care strategies Talk with the patient
**SCHOLAR-MAC** Symptoms Characteristics History Onset Location Aggravating factors Remitting factors Medications Allergies Conditions
**WWHAM** Who is the patient? What are the symptoms? How long have the symptoms been present? Action taken Medications being taken
**SIT DOWN SIR** Site or location of a sign/symptom Intensity or severity Type or nature Duration Onset With (other symptoms) Annoyed or aggravated by Spread or radiation Incidence or frequency Relieved by
**ENCORE** Explore No medication Care Observe Refer Explain

Framework names are shown in bold. The bolded letters within each item correspond to the letters that form the framework mnemonic.

**Table 2:** A priori topic categories derived from prominent OTC counseling frameworks.

	A Priori Categories						
OTC Counseling Framework	Self-Care Appropriateness	Current Medications	Current Health Conditions	Symptom Details	Previous Treatments	Recommendations	Product Details
QuEST	X	X	X	X	X	X	X
SCHOLAR-MAC		X	X	X	X		
WWHAM	X			X	X		
SIT DOWN SIR				X	X		
ENCORE	X	X		X		X	

An “X” indicates that the OTC counseling framework included questions or content related to the corresponding a priori topic category used during deductive content analysis.

**Table 3:** Example questions or statements coded to a priori topic categories.

A Priori Category	Examples
Self-care appropriateness	Are your symptoms severe or getting worse?
Current medications	What prescription or over-the-counter medications are you taking right now?
Current health conditions	Do you have any existing medication conditions?
Symptom details	Can you describe your symptoms to me?
Previous treatments	Have you already tried anything to help your symptoms?
Recommendations	I would recommend using [OTC product] for the symptoms you’re describing.
Product details	You should take this product [dosing instructions] and watch out for [side effects].

**Table 4:** Topic categories discussed during patient–pharmacist consultations (*n* = 32).

Topic Category	Frequency (%)
Product details	28 (88%)
Product recommendation	19 (59%)
Previous treatments	6 (19%)
Current medications	6 (19%)
Current health conditions	6 (19%)
Symptom details	6 (19%)
Self-care appropriateness	1 (3%)
Cost–benefit *	1 (3%)

* the cost–benefit category was identified during inductive content analysis by two independent coders.

**Table 5:** Specific product details discussed during patient–pharmacist consultations (*n* = 28).

Specific Product Detail	Frequency (%) *
Ingredients or drug class	14 (50%)
Dose or frequency	12 (43%)
Side effects	8 (29%)
Mechanism of action	7 (25%)
Indication	6 (21%)
Interactions or contraindications	6 (21%)
Effectiveness	4 (14%)
Dosage forms	2 (7%)
Duration of action	1 (4%)

* frequences of specific product details sum to greater than 28, as some participant–pharmacist interactions included discussions of multiple product details.

**Table 6:** Topic categories discussed during consultations, cough/cold/allergy (*n* = 13) vs. pain (*n* = 17) vs. sleep (*n* = 2) symptom scenario.

Topic Category	Cough/Cold/Allergy Frequency (%)	Pain, Frequency (%)	Sleep, Frequency (%)
Product details	12 (92%)	15 (88%)	1 (50%)
Product recommendation	6 (46%)	11 (65%)	2 (100%)
Previous treatments	2 (15%)	3 (18%)	1 (50%)
Current medications	2 (15%)	4 (24%)	0 (0%)
Current health conditions	2 (15%)	4 (24%)	0 (0%)
Symptom details	2 (15%)	3 (18%)	1 (50%)
Self-care appropriateness	1 (8%)	0 (0%)	0 (0%)
Cost–benefit *	0 (0%)	1 (6%)	0 (0%)

* the cost–benefit category was identified during inductive content analysis by two independent coders.

**Table 7:** Topic categories discussed during consultations, male (*n* = 15) vs. female (*n* = 16) patients *.

Topic Category	Males, Frequency (%)	Females, Frequency (%)
Product details	12 (80%)	15 (94%)
Product recommendation	7 (47%)	11 (69%)
Previous treatments	3 (20%)	3 (19%)
Current medications	2 (13%)	4 (25%)
Current health conditions	1 (7%)	5 (31%)
Symptom details	3 (20%)	3 (19%)
Self-care appropriateness	0 (0%)	1 (6%)
Cost–benefit **	1 (7%)	0 (0%)

* the total sample size is 31 participants, as 1 participant did not complete the follow-up phone interview. ** the cost–benefit category was identified during inductive content analysis by two independent coders.

## Data Availability

The original contributions presented in this study are included in the article. Further inquiries can be directed to the corresponding author.

## Abbreviations

The following abbreviations are used in this manuscript:

OTC	Over-the-counter
JCPP	Joint Commission of Pharmacy Practitioners
ADE	Adverse drug event
MMSE	Mini-Mental State Examination
